# The ferroform and glycoform landscape of human milk lactoferrin dissected through hybrid mass spectrometric approaches

**DOI:** 10.3389/fnut.2025.1697081

**Published:** 2025-12-03

**Authors:** Jing Zhu, Julia Bauzá-Martinez, Joost W. Gouw, Xianfeng Zhao, Albert J. R. Heck, Bernd Stahl, Karli R. Reiding, Kelly A. Dingess

**Affiliations:** 1 Biomolecular Mass Spectrometry and Proteomics, Bijvoet Center for Biomolecular Research and Utrecht Institute for Pharmaceutical Sciences, University of Utrecht, Utrecht, Netherlands; 2 Netherlands Proteomics Center, Utrecht, Netherlands; 3 Institute of Biotechnology and Health, Beijing Academy of Science and Technology, Beijing, China; 4 Skid Visual Science, Ibiza, Spain; 5 Danone Research & Innovation, Utrecht, Netherlands; 6 Health & Science, Open Science Research Center, Danone Nutricia, Shanghai, China; 7 Department of Chemical Biology and Drug Discovery, Utrecht Institute for Pharmaceutical Sciences Utrecht University, Utrecht, Netherlands

**Keywords:** mass spectrometry, glycoproteomics, ion-exchange, human milk, lactoferrin (lf), bioactive protein, native mass spectrometry, proteoform characterization

## Abstract

Human milk lactoferrin (hmLF), a sialic acid-rich iron-binding milk glycoprotein, is essential for infant development, playing a key role in immune defense and gut maturation. Despite its importance, the diversity of hmLF proteoforms, including the relationship between glycosylation profiles (glycoforms) and iron-binding states (ferroforms), has not been systematically characterized. To address this, we used a hybrid mass spectrometry (MS) approach, combining bottom-up glycoproteomics and ion-exchange (IEX) native MS, to analyze hmLF from three human milk donors across lactation. Bottom-up glycoproteomic results revealed that Asn642 remained predominantly not glycosylated, while Asn156 and Asn497 were frequently occupied with biantennary glycans exhibiting variable fucosylation and sialylation. Notably, glycan heterogeneity displayed a site-specific pattern, decreasing consistently in both donors over lactation. Additionally, we used an IEX-native MS strategy to obtain a full picture of the glyco- and ferroforms coexisting in human milk at late lactation stages. IEX separated LF molecules primarily by iron content rather than glycan composition, resulting in five LF fractions with varying iron-loading and glycosylation levels. Native MS full proteoform profiling of these fractions validated that most LF molecules were glycosylated at two of the three available sites, likely Asn156 and Asn497, yet revealed a minor pool of proteoforms glycosylated at all three sites. Careful evaluation of individual fractions and compiled data revealed no clear correlation between ferroform and *N-*glycosylation profiles. These findings provide a detailed overview of hmLF molecular heterogeneity, offering valuable insights for advancing nutritional product development and understanding this bioactive protein’s functional role.

## Introduction

Lactoferrin (LF) is a 78-kDa non-heme iron-binding glycoprotein consisting of 691 amino acids with multiple sialic acid residues attached to *N*-linked glycan chains. Since its discovery in human milk 70 years ago, LF has been also found in other exocrine biofluids such as tears, saliva, semen, nasal, and bronchial secretions (1, 2), as well as an important component of the neutrophilic granules of leucocytes (1, 3, 4). Human milk LF is a multifunctional protein that plays an important role in infant development, by modulating immune defense and gut development (5, 6). Amongst its broad functions, LF shows antimicrobial activity against viruses, bacteria, and fungi (7, 8), displays anti-inflammatory properties (9–12), regulates iron (10, 13) and glucose metabolism (6, 14), promotes bone growth (15), displays antioxidant activity (16–18), and can even inhibit tumor development in some cancers (19–21). These attributes position LF as a key nutritional and bioactive protein, not only in early life, but also in adulthood, as LF is increasingly being explored as a nutritional supplement for its benefits also for adult health (1, 22).

The structure of LF, originally determined in 1987 (1, 23, 24), consists of 2 highly homologous lobes (N- and C-terminal) connected by an alpha-helix peptide. Each of these lobes contains a cleft, capable of chelating one ferric iron ion (Fe^3+^) with high affinity (*K*_D_ ∼ 10^−22^ M) dependent on the concurrent binding of carbonate (CO_2_^3−^) (1, 25, 26). The binding of Fe^3+^ induces a conformational change to a closed, more stable conformation (*holo* LF), while the release of the bound Fe^3+^ reverts LF to a more flexible, open conformation (*apo* LF). Iron loading is assumed to occur in the fully open form of *apo* LF, as unrestricted access allows Fe^3+^ to bind in the clefts. Importantly, despite the high affinity of Fe^3+^-LF interaction, LF can interchange between its *holo* and partly-loaded and *apo* states, where receptor binding or pH reduction promote Fe^3+^ release through opening of the clefts. A key feature that differentiates LF from other members of the transferrin protein family is it can retain Fe^3+^ at pH levels as low as 3–4, a crucial adaptation to the infant’s acidic gut environment (27). In contrast, other members of the transferrin family such as serotransferrin, the release of Fe^3+^ happens already at a pH of 5–6 (27), meaning the iron would be released before being absorbed in the small intestine. Notably, the *apo* and *holo* forms of LF are taken up at similar rates in the small intestine of infants by the LF receptor, although they exhibit differing functionalities once absorbed. *Apo* LF more strongly activates cellular signaling, can bind to the nucleus, and acts as a transcription factor, while *holo* LF is more resistant to degradation (28, 29). Despite the high homology between the N- and C-terminal lobes, some studies have suggested differences in their Fe^3+^ binding affinities and their pH dependency (30).

LF’s functionality is not only related to its ability to bind iron, but also to the *N*-glycans that decorate its surface. While LF bacteriostatic effects are mostly attributed to its ability to sequester free Fe^3+^, starving microbes of a critical nutrient, its bactericidal activity involves disrupting bacterial membranes. This disruption can happen via electrostatic interactions but also via glycosylation-mediated adhesion inhibition (13, 31). There are 3 known *N*-glycosylation sites on human LF (Asn156, Asn497, and Asn642), differing from bovine LF, which contains 5 *N-*glycosylation sites. Typically, only 2 of the 3 hmLF sites are glycosylated (Asn156, Asn497) (32), and these *N*-glycans are comprised of sialylated and fucosylated complex-type structures, many containing Lewis epitopes such as Lewis X (1, 5, 32, 33). Comparative studies indicate that glycosylation patterns in LF differ across species and also among individuals. Barboza et al. (33) demonstrated that glycosylation and glycoforms differ among women, decreasing during the first 2 weeks of lactation, with changes in glycan diversity resulting in a higher degree of fucosylation. Such variations in LF glycosylation have been shown to modulate pathogen interactions and receptor binding, as well as proteolysis resistance (33). Glycosylation of LF has been shown to be highly heterogeneous, not only in humans, but recently also in cow milk (34). This heterogeneity likely introduces variability that can influence pathogen and receptor interactions, as well as resistance to proteolysis.

Understanding these glycoforms, next to the distinct functionalities of *apo* and *holo* LF, is crucial for elucidating LF’s therapeutic potential across life stages. A study by van Berkel *et al*. has shown that the binding of Fe^3+^ was not influenced by glycosylation or lack thereof; however, the study noted that overall resistance to proteolysis of LF was indeed reduced if glycans were removed (22). Additionally, other studies have shown that the iron loading capacity of LF seems independent of glycosylation (1, 22). However, conflicting evidence also reports that de-*N*-glycosylation leads to a great reduction in Fe^3+^ binding capacity of LF (35), as does desialylation (36), when compared to unmodified LF.

In this work, we sought to examine in detail the heterogeneity of human milk LF using hybrid mass spectrometry (MS) approaches. We analyzed a range of samples, including commercially available LF of biological origin (human, bovine) and donor-derived human milk, utilizing both bottom-up and native MS approaches. For the first time, donor-derived hmLF proteoform profile was characterized using native MS, which, in combination with an ion exchange (IEX)-based prefractionation strategy, allowed us to co-resolve iron-loading status, termed ferroform from here onwards, and glycosylation status for each proteoform detected in human milk. This allowed us to interrogate whether any predominant glycoforms were associated with a particular ferroform.

## Materials and methods

### Human subjects and Milk samples

Donors and its demographic data, sample preparation and mass spectrometry (MS) methods have been described in detail previously (37). In short, human milk samples were collected from 2 healthy donors at weeks 1, 2, 3, 4, 6, 8, 10, 12, and 16 postpartum, samples were used for bottom-up glycoproteomics analysis. Human milk from late stage lactation was collected from a third healthy donor for which demographics data was not available. Samples from this third donor were used for IEX-Native MS analysis. Standardized human milk handling conditions (38) were always applied for sample collection. Written informed consent was obtained from all donors prior to collection of samples. All samples were donated to Danone Research & Innovation in accordance with the Helsinki Declaration II.

Next, to donor-derived human milk samples, we also utilized commercially available milk samples from both human and bovine origin. These were commercially available LF purified from human milk (phmLF, L0520, Sigma–Aldrich) and commercially available *holo* (39) LF purified from bovine milk (pbmLF, L9507, Sigma–Aldrich).

### Whole milk proteolytic digestion for glycopeptide analysis

To determine the glycan species, present in hmLF, in-solution digestion of whole milk was performed using an adapted protocol from a previously described method (37, 40). Samples were collected into 2 mL Eppendorf tubes preloaded with protease inhibitors (Complete Mini EDTA-free, Roche) and phosphatase inhibitors (PhosSTOP, Roche), both at a 1/9 ratio of the collection volume. Briefly, 20 μg of proteins were extracted from donor-derived whole milk, pbmLF or phmLF samples in a denaturing buffer containing 1% (*w/v*) sodium deoxycholate (SDC) in 100 mM Tris, and then reduced with 5 mM tris(2-carboxyethyl)-phosphine (TCEP), alkylated with 30 mM 2-chloroacetamide (CAA) at room temperature, and digested with Trypsin (Sigma–Aldrich). Trypsin was added at a 1:50 (*w/w*) enzyme to protein ratio, and samples were incubated overnight (16 h) at 37 °C. SDC was precipitated by acidification with 0.5% trifluoroacetic acid (TFA), and peptides were purified by solid phase extraction using Oasis PRIME HLB 96-well plates (Waters), according to manufacturer’s instructions, dried on a vacuum centrifuge and stored at −80 °C until LC–MS/MS analysis.

### High-pressure liquid chromatography tandem mass spectrometry analysis

Peptides were reconstituted in 2% formic acid (FA), to a volume optimal for achieving an injection of 800 ng of material on the column. All samples were analyzed on an Agilent 1,290 Infinity HPLC system (Agilent Technologies) coupled to an Orbitrap Fusion mass spectrometer (Thermo Fisher Scientific) on 120 min runs. Peptides were first trapped for inside a 100 μm inner diameter, 2 cm trap column (in-house packed with ReproSil-Pur C18-AQ, 3 μm; Dr. Maisch GmbH), before separation on the analytical column (in-house packed with Poroshell 120 EC-C18, 2.7 μm; Agilent Technologies), with a 50 μm inner diameter and 50 cm length. Mobile-phase solvent A consisted of 0.1% FA in water, while mobile-phase solvent B consisted of 0.1% FA on acetonitrile. Trapping was achieved at a flow rate of 5 μL/min for 5 min at 100% A, and peptides were eluted into the analytical column using a passively split flow at 200 nL/min at various percentages of B for the remaining 115 min: peptides were first separated using a linear gradient of 13 to 44% B over 100 min, followed by a steep increase to 100% B over 3 min, 100% B for 1 min, 100 to 0% B over 1 min, and finally the column was re-equilibrated at 100% A for 10 min.

Glycopeptides were monitored without enrichment through bottom-up proteomic analysis using tailored fragmentation strategies that were described in detail before (37, 41). To detect glycopeptides, all samples were run as MS triplicates, each replicate using one of the following 3 fragmentation strategies: higher-energy collisional dissociation (HCD), HCD-product-dependent stepping collision energy HCD (HCD-pd-sHCD) and HCD-product-dependent electron-transfer/higher-energy collision dissociation (HCD-pd-EThcD). For product dependent fragmentation strategies, the triggering ions used are reported in detail in a previous publication (42). Peptides were always ionized on a nano-electrospray ionization source using a 2.0 kV spray voltage. Precursor (MS) scans were acquired within the mass range *m/z* 350–2000, at a mass resolution of 60,000 using an automatic gain control (AGC) target value of 4 × 10^5^ ions in the Orbitrap mass analyzer, or a maximum ion injection time of 50 ms when the AGC target was unmet. Dynamic exclusion was set to 30 s for an exclusion window of 10 ppm with a cycle time of 3 s. Only precursors with 2 + to 8 + charge states and intensities greater than 1 × 10^5^ were selected for fragmentation. Tandem mass spectrometric analysis (MS/MS) was performed on selected precursors using 3 fragmentation strategies. For HCD runs, precursors were isolated using a fixed mass window of 1.6 Th, and fragmentation was performed on the HCD cell using 30% normalized collision energy (NCE). Fragment ions were accumulated to an AGC target value of 5 × 10^4^ or for a maximum injection time of 50 ms, when the AGC target was unmet. Spectra of fragment ions (MS/MS) were acquired on the Orbitrap, within the mass range *m/z* 120–4,000, and at a resolution of 30,000. For product-dependent fragmentation strategies, if at least three oxonium ions of glycopeptides (42) were observed, HCD-pd-sHCD or HCD-pd-EThcD MS/MS on the same precursor was triggered. For HCD-pd-sHCD or HCD-pd-EThcD, precursors were isolated using a mass tolerance of 20 ppm. Product-dependent sHCD was performed at NCEs of 10, 25 and 40%. Product-dependent EThcD was performed at supplemental collision energy of 25%. Fragment ions were accumulated to an AGC target value of 400% the standard value, or for a maximum injection time of 250 ms, when the AGC target was unmet. Spectra of fragment ions (MS/MS) were acquired on the Orbitrap as previously described.

### Glycopeptide identification

For glycopeptide identification, all the obtained “raw” files were processed in Byonic (Protein Metrics Inc., v. 3.9.4, Cupertino, CA, USA) and searched against a database containing human F protein (UniProtKB accession P02788, downloaded 22 September 2020), or bovine LF protein (UniProtKB accession P24627, downloaded on 22 September 2020) with the following search parameters: tryptic digestion, with precursor ion mass tolerance of 10 ppm, fragmentation type including both HCD and EThcD, and fragment mass tolerance of 20 ppm; Carbamidomethylation of cysteines (+57.02 Da) was set as a fixed modification, while variable modifications included methionine oxidation (+15.99 Da, rare), phosphorylation of serine, threonine, and tyrosine (+79.97 Da, common), and glutamine to pyro-glutamate conversion (−17.03 Da, rare) as well as glutamate to pyro-glutamate conversion (−18.01 Da, rare) both at the N-terminus or protein N-terminus. For glycan analysis, we used a Byonic database containing 279 N-glycans, thoroughly detailed in a previous publication (42). From the curated list of glycans, we acknowledge 2 limiting factors of our analytical approach: first, as we collected compositional information, we could not distinguish between isomeric monosaccharides (such as GalNAc and GlcNAc residues) and second, peptides which have been assigned with multiple *N-*glycans could also be assigned as carring one large glycan or a mixture of these mass-matching possibilities instead. The maximum number of precursors per scan was set to 1, and the FDR at 1%. Data was further curated by non-negligible error probabilities |log prob.| ≥ 1.5, score ≥ 150, and delta mod score ≥ 10 was considered acceptable. Additionally, the signal peptide was removed from the full protein sequence. The remaining reverse hits (<1%) were also removed before subsequent data analysis.

### Processing of phmLF and pbmLF samples prior to IEX chromatography and native MS analysis

To determine whether iron-loading or glycan composition were impacting separation on IEX chromatography, the phmLF sample was preprocessed to generate suitable standards. Iron-loading and desialylation treatments were performed on phmLF as follows: 200 μg of phmLF were loaded with iron (Fe) as previously described (43), although using the naturally available Fe isotopes (being ^56^Fe the predominant one) instead of the heavy ^59^Fe isotope. Briefly, FeCl_3_ was chelated to a 5-fold molar excess of nitrilotriacetate (NTA) and then incubated at a 2:1 molar ratio of Ferric-Nitrilotriacetate (Fe-NTA) with phmLF at 4 °C overnight. The sample was then buffer exchanged into 100 mM Ammonium Acetate (AMAC) in water by ultrafiltration (Amicon, Merck) using a 10 kDa cutoff filter. The latter step removed non-chelate iron ions and concentrated the protein. For enzymatic sialic acid removal, phmLF was dissolved in 100 mM AMAC and treated with sialidase (neuraminidase *from Arthrobacter ureafaciens*, 10,269,611,001, Roche) at an enzyme-to-protein ratio of 0.06 U:50 μg, followed by incubation at 37 °C for 4 h. These samples were then further processed on the IEX chromatography, as described in the following section, with the objective of assessing peaks’ shift based on the abovementioned treatments. However, due to high purity of phmLF sample, no fractionation was performed.

Processing of pbmLF was achieved by resuspending 50 μg in Phosphate Saline Buffer (PBS), and buffer exchanged into 150 mM AMAC in water using a 10 kDa cutoff filter. In this case, IEX was not performed and processed pbmLF samples were directly analyzed by high-resolution native MS (44).

### Dual-column IEX chromatography separation of unprocessed human milk prior to native MS analysis

For IEX, we used an adapted Agilent 1,290 Infinity HPLC system (Agilent Technologies, equipped with a tandem WAX-CAT two-stage set-up (45) (PolyLC Inc.) that has been described in detail, both in terms of operation and components, in a previous publication (46). To remove the lipidic component, whole milk was processed by centrifugation at 10,000 × g for 30 min at 4 °C, with skim milk being obtained. For each skim milk sample analyzed, about 250 μg total protein (according to the known protein concentratoin of skim milk) were loaded in the system. Briefly, the system consisted of a dual-column tandem WAX-CAT two-stage set-up, consisiting of a PolyWAX LP column (200 × 2.1 mm i.d., 5 μm, 1,000 Å, from PolyLC Inc) and a PolyCAT A column (50 × 2.1 mm i.d., 5 μm, 1,000 Å, from PolyLC Inc). Columns were refrigerated at 17 °C while other compartments were kept at 4 °C to minimize sample degradation. Elution was achieved using a 5-step gradient using incremental percentage of mobile-phase buffer B. Briefly, system was equilibrated at 0% B for 6 min, then proteins were eluted for 36 min using a salt gradient of 0–60% B, then the columns were rinsed for 18 min using high salt gradient of 60–100% B, then a high-salt wash was performed on the system for 1 min at 100% B before dropping to 0% B for system restoration before the end of the run. Mobile phase buffer B consisted of 2.5 M AMAC in water, while mobile phase buffer A consisted of 100 mM AMAC in water. To minimize microbial contamination, low concentration NaN3 (3 mM) was added to buffers, and these were filtered using a 0.22 μm cartridges (Millipore) before use. The flow rate was set to 800 μL/min. Chromatograms were monitored at 280 nm and peak-based fractions of 1 mL were collected using an automated fraction collector.

### Native MS analysis

IEX fractions from all donor-derived hmLF samples, as well as for phmLF and pbmLF samples, were analyzed individually on a modified Q Exactive Plus Orbitrap instrument with ultra-high mass range (UHMR) (Thermo Fisher Scientific). Spectra were recorded using *m/z* range of 500–10,000. The voltage offsets on the transport multi-poles and ion lenses were manually tuned to achieve optimal transmission of protein ions at elevated *m/z*. In the HCD cell, nitrogen was used as a gas, at a pressure of 6–8 × 10^−10^ bar, and at a fragmentation energy of 50 V. Spray voltage was set to 1.2–1.3 V, in-source fragmentation was set to 30 V, source temperature was set to 250 °C, and all spectra were recorded at a resolution 17,500 at *m/z* 200. Mass calibration of the instrument was achieved using a solution of CsI as previously described (47).

### Native MS data analysis

For all observed proteoforms of hmLF, accurate masses were calculated manually for the most abundant charge state: 19 + for hmLF and 18 + for pbmLF. The average mass of hmLF, including disulfide bonds and excluding the signal peptide, was calculated to be 76,132.99 Da, and for pbmLF, the average mass was calculated to be of 76,111.60 Da. To determine the glycans species present in hmLF and pbmLF proteoforms, data were manually processed, and glycan structures were deduced based on the evidence obtained on the previously described glycoproteomic analysis as well as on known biosynthetic pathways. The following average masses were used to assign glycan moieties: hexose (i.e., Glucose, Glc; mannose, Man; Galactose, Gal; 162.1424 Da), *N*-acetylhexosamine (HexNAc, i.e., GlcNAc or GalNAc; 203.1950 Da), Fucose (dHex, 146.143 Da), and *N*-acetylneuraminic acid (NeuAc, 291.2579 Da). The average mass of ferric iron ion (Fe^3+^, 55.845 Da) and of hydrogen (H^+^, 1.0079 Da) were used. The molecular weight of each peak in the spectra was calculated using the formula:


$${\mathrm{MW}}_{\text{measured}}=(m/z_{\text{measured}}\;x\;z)–[(z–n\times 3)\times H^{+}],$$

where *z* is the charge, *n* is the number of bound Fe^3+^, and H^+^ is the mass of hydrogen.

### Data processing, visualization, and availability

Data processing and visualization was done using Excel and R Studio (version 2022.10), using R (version 4.2.2). Figures were edited using Adobe Illustrator (version 28.0). Structural representations of hmLF were done using Pymol (version 2.4.1, Schrödinger) and are based on the high-resolution crystal structure of hmLF deposited in the Protein Data Bank entry 1FCK (PDB Entry:1FCK) (48). All symbols and text nomenclature used are according to recommendations of the Consortium for Functional Glycomics (49). The MS raw data generated in this study has been deposited in the ProteomeXchange partner MassIVE database under the accession code MSV000097762 (50). Deposited data include bottom-up glycoproteomics and native MS ‘.raw’ files. Data supporting the findings presented in this manuscript can be made available upon request to the corresponding author.

## Results

### Assessing site-specific longitudinal trends in the glycosylation of human milk derived LF by bottom-up glycoproteomics

**Figure 1:**
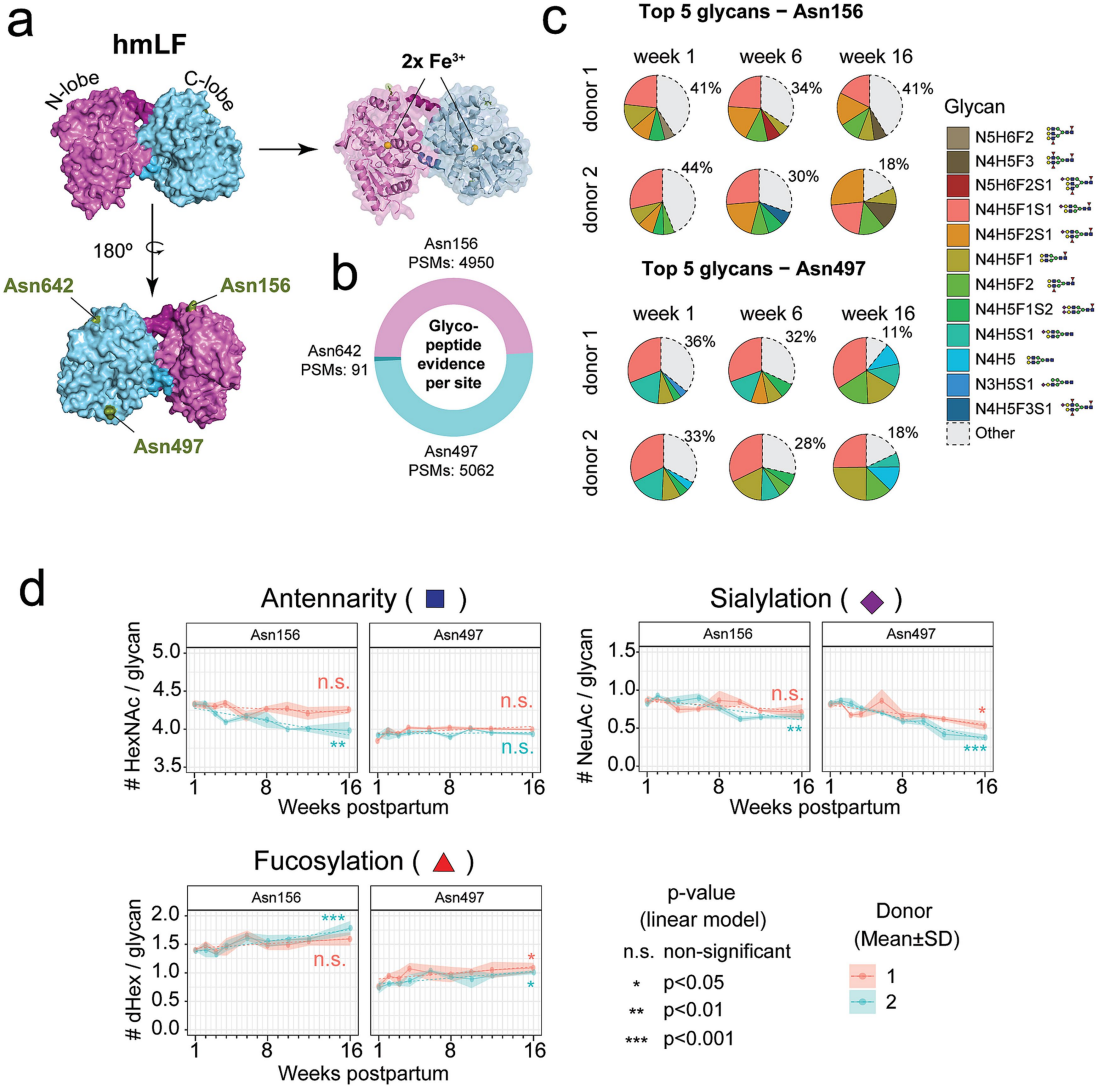
Bottom-up glycoproteomics analysis reveals only marginal longitudinal changes in the glycan composition of human milk LF (hmLF). **(a)** Structural model of human LF (front and back views). Relevant structural features are annotated in pink and blue, and the 3 known *N*-glycosylation sites are annotated in green. **(b)** The cumulative evidence, as represented by the total number of (glyco)peptide spectrum matches (PSMs), observed for each of the glycosylation sites, i.e., Asn156, Asn497 and Asn642, is displayed. Color corresponds to the lobe in which the glycosylation site is located. **(c)** The top 5 most abundant glycan moieties observed for each donor at weeks 1, 6 and 16 are displayed for sites Asn156 (top) and Asn497 (bottom). The percentage represented by the “Other” fraction is displayed. **(d)** Antennarity (i.e., number of HexNAcs per glycan), Fucosylation (i.e., number of dHex per glycan) and Sialylation (i.e., number of NeuAc per glycan), are shown for sites Asn156 (left) and Asn497 (right) for both donors. *p*-values for linear models (dashed lines) performed over lactation are displayed for each donor and site. Data points represent the average of 3 replicate injections, and positive/negative standard deviation are displayed as a ribbon.

**Figure 2:**
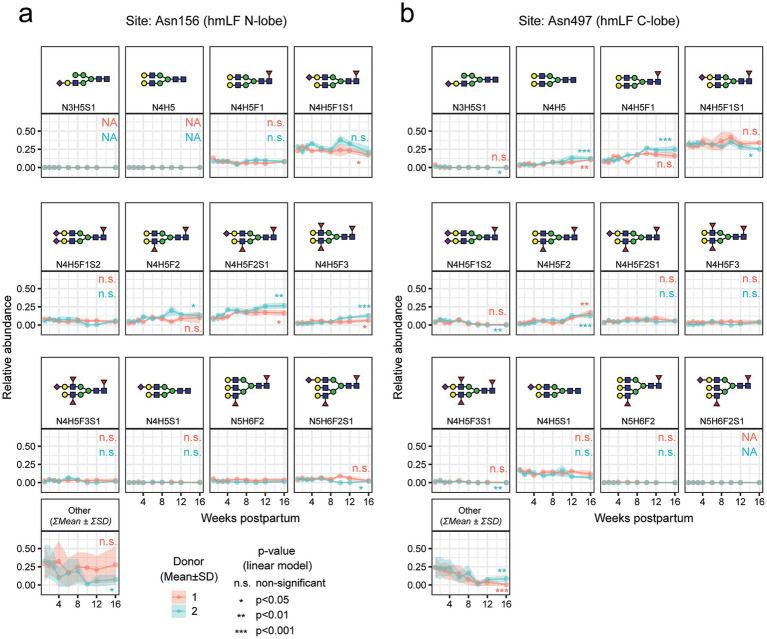
Longitudinal trends of total hmLF abundance as well as for the top 5 most abundant glycan moieties found for both donors at sites Asn156 and Asn497 of hmLF, as measured by bottom-up glycoproteomics analysis (37). **(a)** Asn156 and **(b)** Asn497 derived glycan moieties longitudinal trends, for all the glycans displayed on Figure 1c, i.e., the top 5 most abundant on either week 1, 6 or 16. Data points represent the average of 3 replicate injections, and positive/negative standard deviation are displayed as a ribbon. For all other glycan species detected within the “other” portion of the pie charts displayed in Figure 1c, the sum of the mean of the 3 replicates is taken.

To comprehensively characterize the *N*-glycosylation of hmLF longitudinally over lactation, we first applied a bottom-up glycoproteomics approach to milk-derived LF from 2 donors collected over the first 16 weeks postpartum. Structurally, hmLF consists of two lobes (N- and C-terminal), each capable of binding one Fe^3+^ ion. As shown in Figure 1 the three reported *N*-glycosylation sites – Asn156, Asn497, and Asn642 – are located on the “back side” of the protein. These sites are not evenly distributed between the 2 lobes: the N-lobe contains Asn156, while the C-lobe contains both Asn497 and Asn642 (Figure 1a). First, bottom-up glycoproteomics identified multiple glycosylated Peptide Spectrum Matches (PSMs) of all 3 *N*-glycosylation sites, which also provided indications of glycan occupancy. However, the site Asn642 showed limited spectral evidence, suggesting low site occupancy (Figure 1b). Focusing on sites Asn156 and Asn497, for which thousands of glycosylated PSMs were obtained for each site (Figure 1b), we could identify a diverse repertoire of glycan structures decorating them. Despite the observed wide diversity, a small subset of predominant glycans was consistently present on these sites, with markedly similar profiles between both donors (Figure 1c). The relative abundance of these predominant glycans remained relatively stable, although their dominance increased slightly over lactation time, as reflected in the decreasing proportion of glycans categorized as “others” (Figure 1c, gray-dashed, and Figures 2a,b). To further investigate whether specific glycan features were correlated with these sites, we analyzed the features antennarity (i.e., branching), sialylation, and fucosylation across the lactation period (Figure 1d). Antennarity remained consistent across sites and donors. Sialylation slightly decreased, and fucosylation slightly increased over lactation at Asn497 (Figure 1d), although these trends were not always statistically significant between both donors (*n.s.,* linear model *p*-value).

### Setting up a detailed protein-centric approach to profile ferroforms and glycoforms in human milk LF using IEX coupled with native MS

While bottom-up glycoproteomics provides a complete picture of the glycans decorating the *N-*glycosites of hmLF, this approach falls short in capturing the complete quantitative picture of co-existing glycoforms present in human milk. To address this limitation, we performed a native MS analysis of milk-derived LF to obtain a comprehensive view of its proteoform landscape (51). Given the intrinsically high heterogeneity of LF, as recently demonstrated for bovine milk-derived LF also using native MS (34) and for hmLF in terms of glycan complexity (Figure 1) by our work and that of others (32, 33), we expected a complex proteoform profile upon Native MS analysis. Aiming to handle the expected proteoform complexity, we incorporated an IEX-based prefractionation strategy, which has been shown to enhance proteomic detection and to improve the resolution of complex proteoform profiles (45, 52).

**Figure 3:**
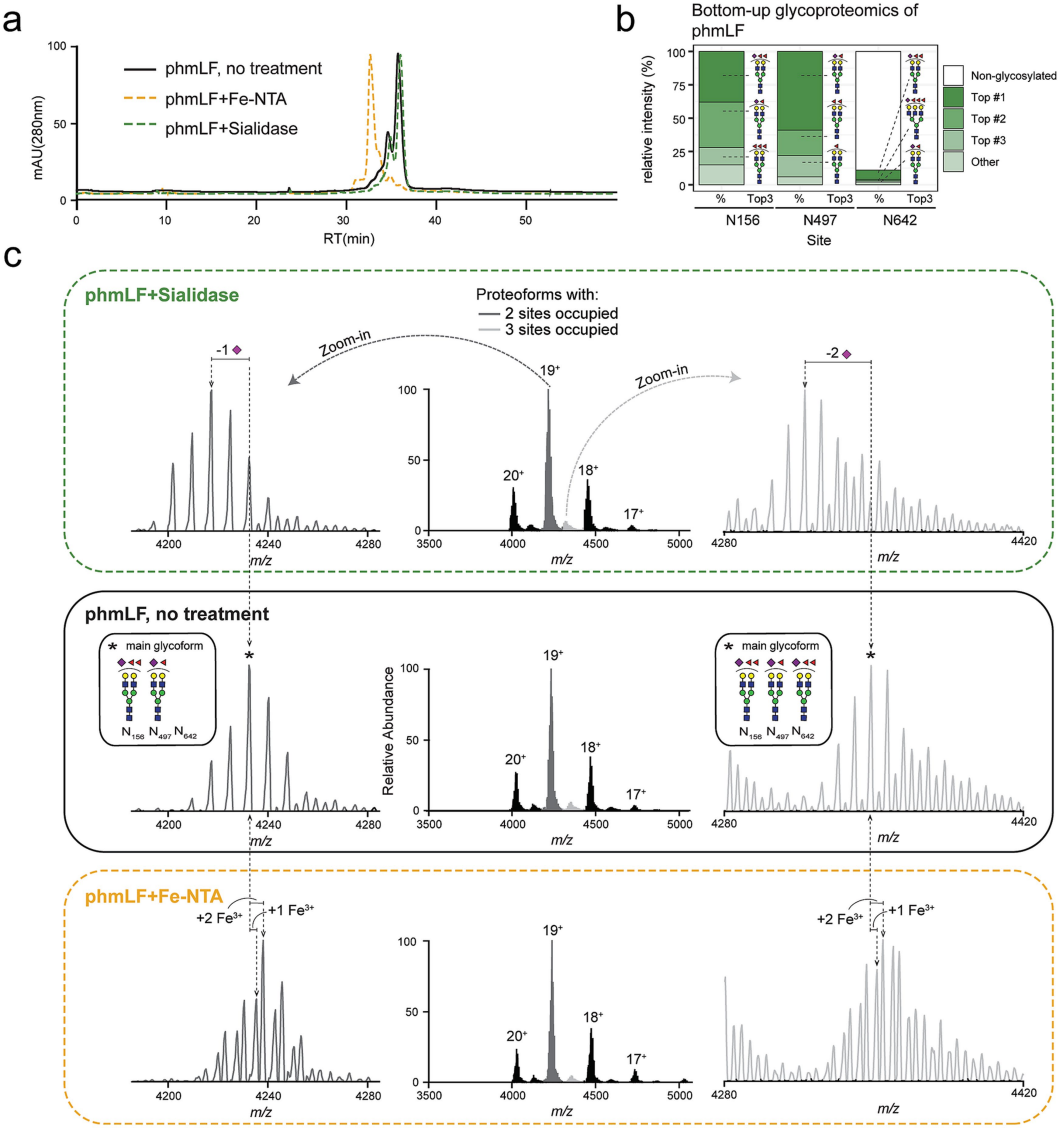
LF proteoforms separate primarily based on Fe3 + content in IEX chromatography. **(a)** The elution profile of intact purified human milk LF (phmLF, black), iron-saturated phmLF as achieved by Fe-NTA treatment (yellow) and desialylated phmLF as achieved by treatment with sialidase (green) is shown. Separation over time (min.) is performed in a dual-column IEX chromatography system and response is measured using mAU (280 nm). **(b)** The relative abundance (%) for the most abundant glycoforms in each glycosylation site (Asn156, Asn497, and Asn642) on the untreated phmLF sample is displayed. Relative abundance is measured at glycopeptide level, in an independent bottom-up glycoproteomic analysis. The average relative abundance of 2 replicate injections is shown. Beyond the top 3 most abundant glycans, all other glycan species detected are summarized under “other” category. Relative abundance of non-glycosylated peptide counterparts is shown. **(c)** High-resolution native MS spectrum for untreated phmLF (black, middle), phmLF treated with sialidase (green, top) and phmLF treated with FeNTA (yellow, bottom). For the 2 main proteoform clusters, i.e., likely glycosylated only at sites Asn156 and Asn497 (dark gray) or glycosylated at all 3 sites (light gray), the base peak is marked with an asterisk and the most abundant glycan species found for each site was used to calculate the exact mass. Mass shifts corresponding to sialic acid removal (−291 Da, or equivalent −15.33 *m/z* for 19+) or Fe3 + addition (+56 Da, or equivalent +2.93 *m/z* for 19+) are indicated. An insert is included showing the glycans annotated for the base peak (marked with an asterisk).

To benchmark IEX separation behavior, we prepared 3 variants of a commercially available sample of human-milk derived purified LF (phmLF): untreated phmLF, iron-saturated phmLF (obtained by Fe-NTA treatment), and desialylated phmLF (obtained by enzymatic treatment with a sialidase). The elution profile obtained upon IEX chromatography performed on these 3 samples indicated that separation was influenced mainly by iron-loading status. Iron-saturated phmLF eluted earlier (Figure 3a, yellow trace) compared to the non-saturated untreated phmLF sample (Figure 3a, black trace) and to the non-saturated desialylated phmLF samples (Figure 3a, green trace). Sialylation did not seem to significantly alter separation, since the chromatographic trace of desialylated phmLF (Figure 3a, green trace) highly overlapped with that of untreated phmLF (Figure 3a, black trace).

To validate these findings, we then applied native MS and bottom-up glycoproteomics to these samples. Bottom-up glycoproteomics confirmed that the most abundant glycans in the independent phmLF sample (Figure 3b) aligned well with the top 5 most abundant glycans identified in donor-derived hmLF at sites Asn156 and Asn497 (Figure 1c). Additionally, Asn642 was indeed largely unoccupied (Figure 3b), consistent with donor-derived hmLF glycopeptide spectral evidence (Figure 1b). Native MS analysis of untreated phmLF revealed 2 major clusters of proteoforms: a larger cluster (Figure 3c, dark gray, > 90% relative abundance) corresponding to proteoforms glycosylated at 2 of 3 *N*-glycosylation sites, and a smaller cluster (Figure 3c, light gray, < 10% relative abundance) corresponding to hmLF glycosylated at all 3 sites. Differences in *m/z* values were diagnostic of specific features, including the expected loss of sialic acid upon desialylation, or the gain of 1 or 2 Fe^3+^ ions upon Fe-NTA treatment. The most abundant glycoform in the untreated phmLF sample (Figure 3c, black-enclosed panel, base peak labelled with an asterisk) contained a combination of glycans commonly observed on hmLF glycosites (Figure 3b and Figure 1b).

**Figure 4:**
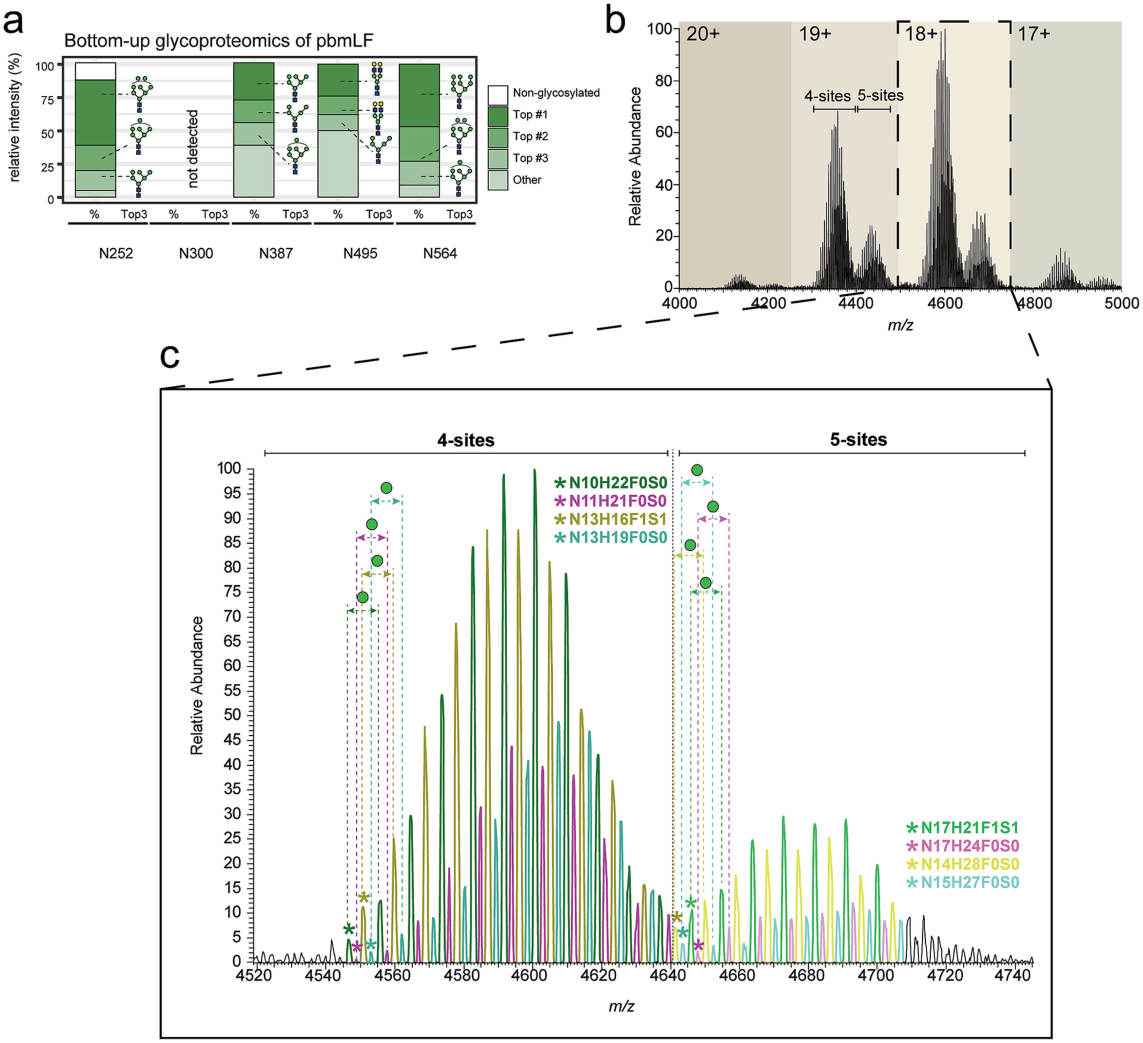
Native MS charaterization of purified bovine milk LF (pbmLF) glycoform and ferroform profiles. **(a)** The relative abundance (%) for the most abundant glycoforms in each glycosylation site (Asn252, Asn387, Asn495, and Asn564) on the purified pbmLF sample is displayed. Relative abundance is measured at glycopeptide level, in an independent bottom-up glycoproteomic analysis. The average relative abundance of 2 replicate injections is shown. Beyond the top 3 most abundant glycans, all other glycan species detected are summarized under “other” category. Relative abundance of non-glycosylated peptide counterparts is shown. **(b)** Native mass spectrum of the purified pbmLF sample revealed two large proteoform clusters at each charge state, corresponding to pbmLF glycosylated at 4 *N*-glycosylation sites or pbmLF glycosylated at all 5 sites. **(c)** For the most abundant charge state (*z* = 18+), a zoom-in to peaks in both proteoform clusters is provided. For these proteoforms, all peaks correspond to holo pbmLF. For all peaks, a combined glycan formula was calculated based on the glycan composition of individual glycans most probably present at each site. For the first peak (asterisk) within each proteoform distribution (as defined per peak color), the combined glycan formula is given. Since mass differences between peaks of the same color were always constant and equivalent to a hexose gain, this helps the viewer obtain the combined glycan composition of any other peak in the spectrum. Mass shifts corresponding to one hexose are highlighted for the 4 glycoform distributions (±162 Da, or equivalent ±9 *m/z* for 18+).

Recently, bovine milk-derived LF was also characterized using a native MS approach (34), providing novel insights into its heterogeneity and glycosylation profiles. Here, we also analyzed a commercially available LF sample purified from bovine milk (pbmLF) using our hybrid MS approach to validate and extend these findings. First, glycoproteomic analysis mapped glycans at 4 of the 5 *N*-glycosites present in bovine LF (Figure 4a), revealing predominantly high-mannose glycans at Asn252, Asn387, and Asn564, and relatively simple complex-type glycans at Asn495. Data for Asn300 was unavailable due to proteolytic inaccessibility (using trypsin and AspN) of glycopeptides for this site. However, literature reports this site to contain complex-type glycans (53) with high sialylation frequency and to be less frequently glycosylated than other pbmLF glycosites (34, 54). Notably, pbmLF also displayed two major proteoform clusters, one glycosylated at 4 out of 5 sites and another, less abundant, representing fully glycosylated proteoforms, the occurrence of which was also previously reported (55). However, fully glycosylated pbmLF proteoforms were more prevalent (20–25% relative abundance) than what observed in phmLF (< 10%, Figure 3b). Carefull examination of the 18 + charge state (Figure 4c) revealed mostly high-mannose type glycoforms with varying degrees of mannosylation, consistent with the glycan heterogeneity recently reported (34). Finally, all pbmLF proteoforms detected were mapped to the fully iron-saturated ferroform, i.e., *holo* pbmLF, since we could not detect any mass differences corresponding to the loss of Fe^3+^ ions in this sample.

### Application of IEX-native MS for in-depth profiling of ferroforms and glycoforms directly from donor-derived human milk LF

**Figure 5:**
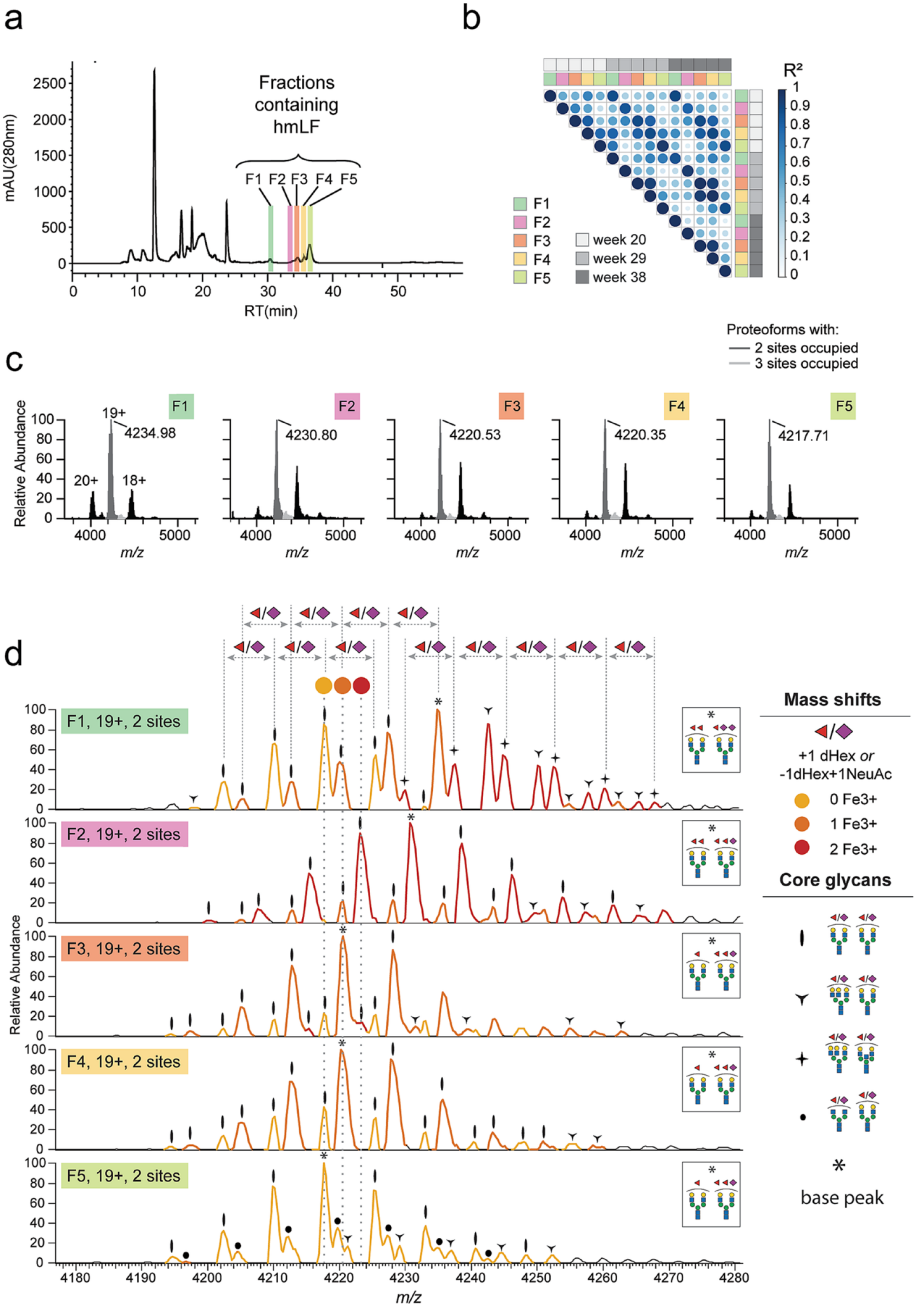
IEX-native MS analysis of human milk LF (hmLF) on donor-derived delipidated milk. **(a)** The IEX chromatography elution profile of delipidated human milk obtained from a donor at week 29 postpartum. Peak identity was determined using independent bottom-up proteomics and fractions where hmLF was eluting were separated and are highlighted as F1-F5 over the elution profile. Response is measured using mAU (280 nm). **(b)** Native MS analysis was performed on each fraction, and Pearson’s correlation of the extracted masses for each lactation time-point and each fraction is displayed. A Pearson’s correlation matrix is displayed for fractions eluted from milk derived from this donor at weeks 20, 29 and 38 postpartum. Within each fraction, R2 remained very high independently of the sampling week, while different fractions displayed very low correlation. **(c)** Native mass spectrum of each hmLF sample revealed two main proteoform clusters at each charge state, corresponding to hmLF glycosylated at 2 *N*-glycosylation sites (dark gray) or at all 3 sites (light gray). **(d)** For the most abundant charge state (*z* = 19+), a zoom-in to peaks in the large proteoform cluster (dark gray, glycosylated at 2 sites) is provided. For these proteoforms, all peaks correspond to hmLF proteoforms with a varying degree of iron-loading and a varying glycosylation profile. Regarding iron-loading status, later-eluting fractions contained a higher proportion of apo hmLF and earlier eluting fractions contained a higher proportion of holo hmLF, except for F1 which displayed a more heterogeneous ferroform profile. For each peak of these doubly glycosylated proteoforms, a symbol corresponding to the annotated core glycan structure is displayed. Within each proteoform distribution, the core glycans were found with a varying degree of fucosylation and sialylation, as reflected by the annotated mass shifts (±146 Da, or equivalent ±7.6 *m/z* for 19+) consistently found between adjacent. Ferroforms, differing by 2.93 *i.w.,* for 19+, are indicated by peak color (yellow for apo hmLF, orange for partly loaded hmLF and red for holo hmLF). Finally, base peaks, i.e., the most abundant proteoform, are annotated over the spectrum with an asterisk, and the complete glycoform annotated for it is displayed in an insert right of the spectrum.

To obtain a complete picture of the heterogeneity and iron-loading status of hmLF, we then applied this IEX-native MS strategy to milk obtained from a human donor. For this analysis, and considering the decreasing heterogeneity observed for glycoforms along milk maturation (Figure 1c), we decided to use mature human milk samples to facilitate mass annotations – i.e., weeks 20, 29, and 38 postpartum. For these timepoints, we injected delipidated donor-derived milk into our IEX setup to achieve fractionation of hmLF proteoforms. Upon IEX separation, 5 fractions containing hmLF were obtained, as confirmed by conventional bottom-up proteomic analysis, and these were labelled F1-F5 (Figure 5a). Moreover, chromatographic profiles and intensity-based absolute quantification (iBAQ) values displayed a very high correlation (Pearson’s correlation R^2^ = 0.997) between repeated IEX measurements, indicating a very high reproducibility of IEX separation (*data not shown*). Upon identification of the hmLF-containing fractions, native MS analysis was performed individually on each fraction. The extracted masses for each fraction were contrasted for the 3 lactation timepoints, revealing a very high correlation in the proteoform profiles present within one fraction for the 3 timepoints analyzed (Figure 5b). This indicated that the driver of differences in the proteoform profiles in mature milk were mainly the IEX fractions rather than the sampling week (Figure 5b). Therefore, we focused our analysis on the fractions corresponding to the mid timepoint (week 29).

First, in agreement with prior analysis of phmLF by native MS (Figure 3c), the predominant charge state was consistently 19 + across all fractions (Figure 5c), and the predominant peak cluster corresponded again to hmLF glycosylated at 2 of the 3 *N*-glycosylation sites (Figure 5c, dark gray). In fact, fully glycosylated hmLF represented less than 5% of all proteoforms measured (Figure 5c, light gray). Focusing on the major proteoforms, which are glycosylated at 2 sites, we assigned the iron-loading status, i.e., ferroforms, or LF loaded with either 0, 1, or 2 Fe^3+^ ions. We also assigned the glycan masses to all well-resolved peaks measured in each fraction (Figure 5d) to determine the glycoforms present in the sample. Except for fraction F1, which displayed a more heterogeneous glyco- and ferroform profile, but also eluted significantly earlier in the IEX system (Figure 5a), all the other fractions separated preferentially specific ferroforms (Figure 5d). Specifically, fraction F2 predominantly separated *holo* hmLF ferroforms (red peaks), fraction F3 and F4 separated partly loaded LF ferroforms (orange peaks), and fraction F5 separated mainly *apo* hmLF ferroforms (yellow). In terms of glycoforms, for these 2-site glycosylated proteoforms, both core glycans were predominantly diantennary with various degrees of fucosylation and sialylation, regardless of the iron-loading status. However, glycoforms with a bisecting diantennary, and/or a triantennary core glycan were also detected with varying amounts of fucosylation and sialylation and diverse iron-loading statuses. This information is detailed in Figure 5d, where the ferroform (iron-loading status) is denoted by the peak color and annotated mass shift (±56 Da, or equivalent ±2.93 *m/z* for 19+), the main core glycans are denoted by the symbols over each peak, and the varying degree of fucosylation and sialylation is evidenced by the annotated mass shifts (±146 Da, or equivalent ±7.6 *m/z* for 19+) consistently found between adjacent peaks belonging to the same proteoform distribution (i.e., same glycan core and ferroform). In Figure 5d, the base peak, i.e., the most abundant proteoform within each fraction, is labelled with an asterisk. For the base peaks, the glycoform that best explains its measured mass is displayed in an insert to the right of each fraction’s spectra (Figure 5d, insert) and strongly agrees with the most abundant glycans expected for hmLF (Figures 1c, 3b).

**Figure 6:**
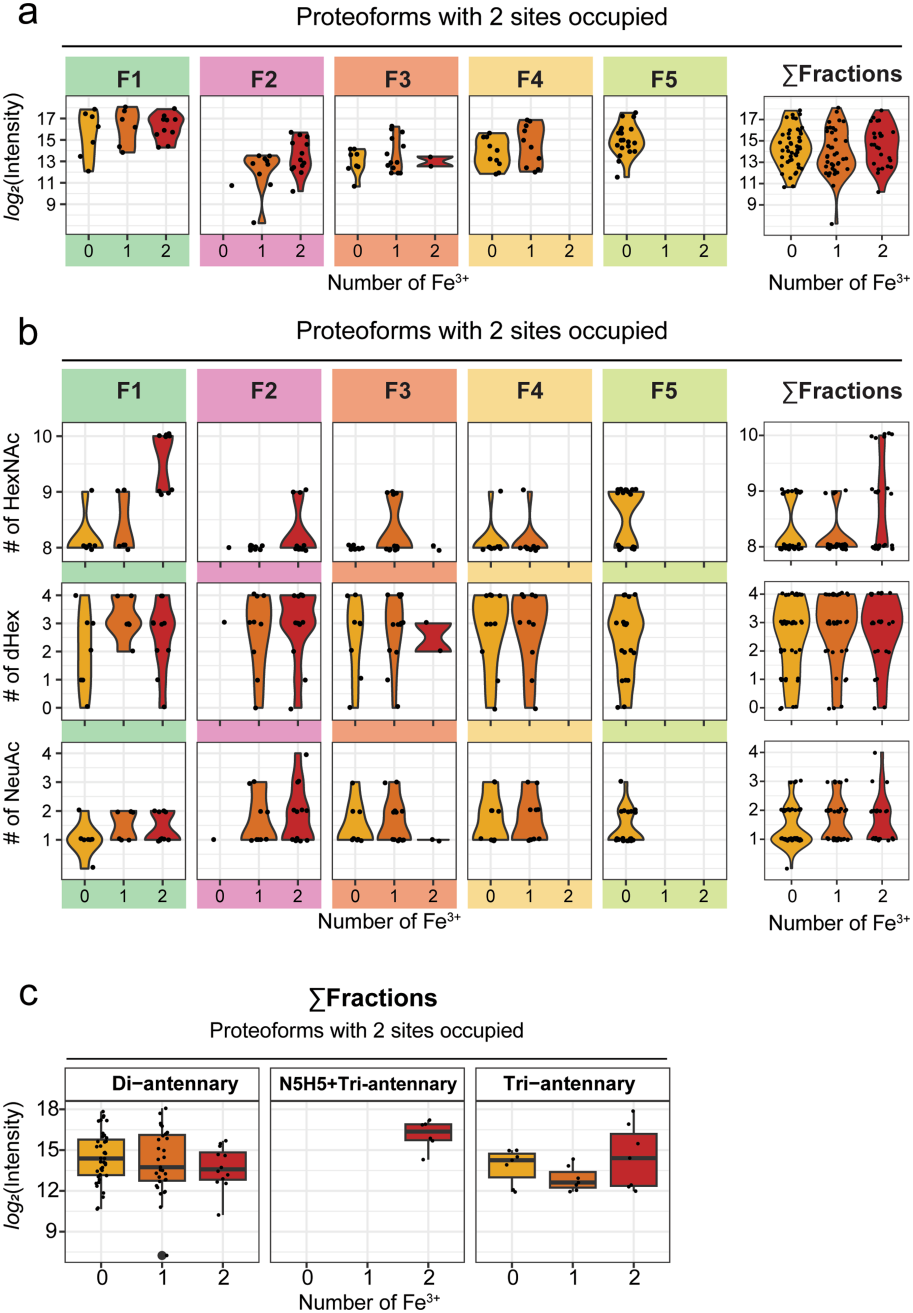
Analysis of ferroform-glycoform interplay for human milk LF (hmLF) proteoforms with 2 out of 3 *N*-glycosylation sites occupied. **(a)** For each annotated ferroform peak (yellow for apo hmLF, orange for partly loaded hmLF and red for holo hmLF), *log_2_* intensity was extracted and plotted either for peaks within each fraction or for the total proteoforms detected independently of the fraction of elution (collided data, *Σ* fractions). Each proteoform, i.e., an annotated peak in the spectrum, is displayed as black dot, and violin plots are used to show the distribution around each ferroform. **(b)** For each well-resolved peak, a combined glycan formula was calculated based on the individual glycans best explaining its mass. The number of *N*-acetylhexosamines (HexNAcs), Fucoses (dHex) or Sialic acids (NeuAc) of the resulting combined glycan formula is shown at ferroform level, plotted either for peaks within each fraction or for the total proteoforms detected independently of the fraction of elution (collided data, Σ fractions). Each proteoform, i.e., an annotated peak in the spectrum, is displayed as black dot, and violin plots are used to show the distribution around each ferroform. **(c)** The boxplots, from left to right, display *log_2_* intensity for ferroforms whose core contains only di-antennary glycans, or one bisecting (N5H5) + one triantennary glycan, or at least one tri-antennary glycan. Data is displayed for the total proteoforms detected independently of the fraction of elution (collided data, Σ fractions). Each proteoform, i.e., an annotated peak in the spectrum, is displayed as black dot. Boxplots display the median (central line), interquartile range (IQR) (box from Q1 to Q3), and whiskers extending to 1.5 × IQR. Outliers are plotted beyond whiskers (thicker dots).

**Figure 7:**
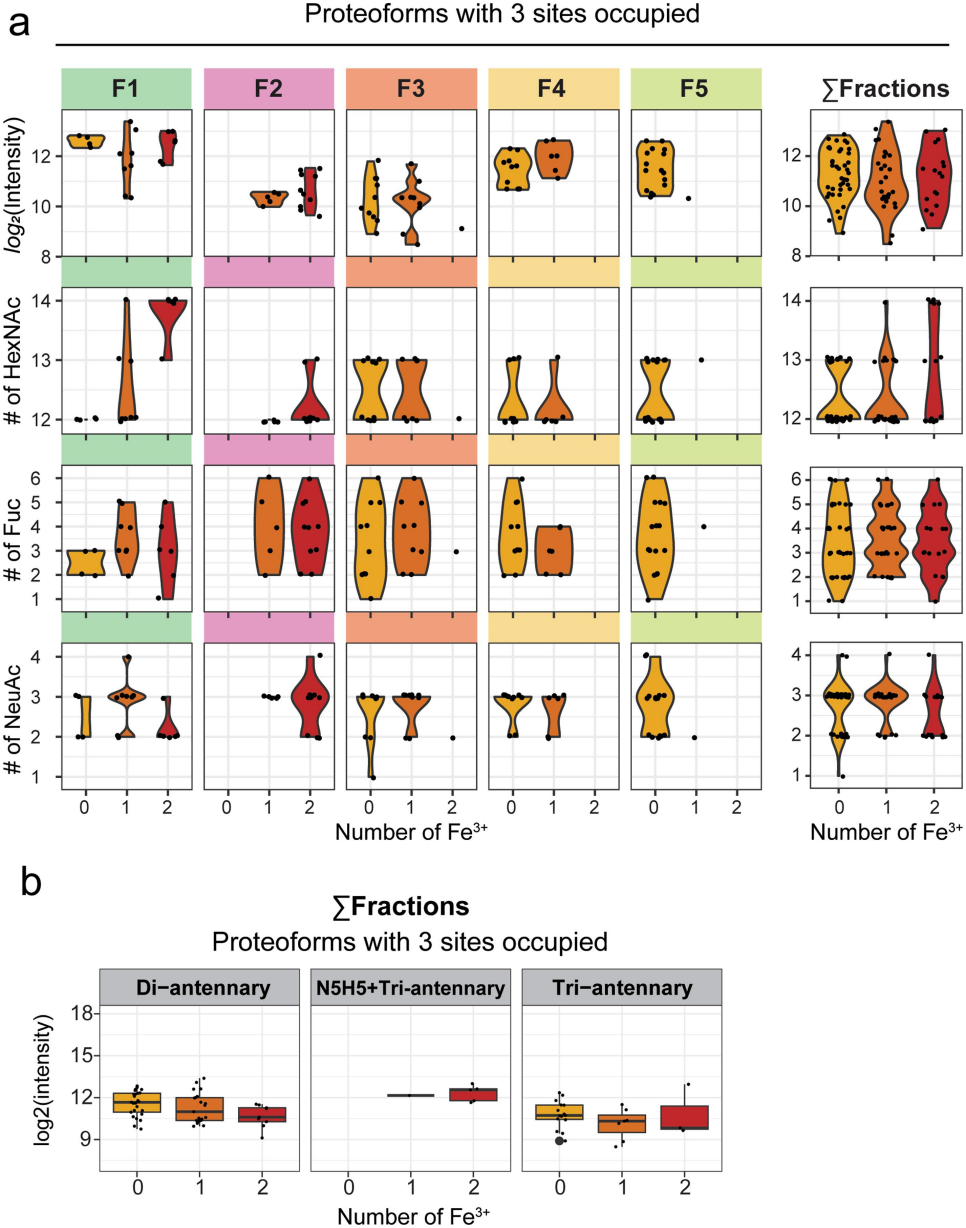
Analysis of ferroform-glycoform interplay for fully glycosylated human milk LF (hmLF) proteoforms. **(a)** For each annotated ferroform peak (yellow for apo hmLF, orange for partly loaded hmLF and red for holo hmLF), *log*_2_ intensity was extracted and plotted either for peaks within each fraction or for the total proteoforms detected independently of the fraction of elution (collided data, Σ fractions). Each proteoform, i.e., an annotated peak in the spectrum, is displayed as black dot, and violin plots are used to show the distribution around each ferroform. For each well-resolved peak, a combined glycan formula was calculated based on the individual glycans best explaining its mass. The number of N-Acetylhexosamines (HexNAcs), Fucose (dHex) or Sialic acid (NeuAc) of the resulting combined glycan formula is shown at ferroform level, plotted either for peaks within each fraction or for the total proteoforms detected independently of the fraction of elution (collided data, Σ fractions). Each proteoform, i.e., an annotated peak in the spectrum, is displayed as black dot, and violin plots are used to show the distribution around each ferroform. **(b)** The boxplots, from left to right, display log2 intensity for ferroforms whose core contains only di-antennary glycans, or one bisecting (N5H5) + one triantennary glycan, or at least one tri-antennary glycan. Data is displayed for the total proteoforms detected independently of the fraction of elution (collided data, Σ fractions). Each proteoform, i.e., an annotated peak in the spectrum, is displayed as black dot. Boxplots display the median (central line), interquartile range (IQR) (box from Q1 to Q3), and whiskers extending to 1.5 × IQR. Outliers are plotted beyond whiskers (thicker dots).

Second, given the resolution that this IEX-Native MS approach can provide into the proteoform landscape of donor-derived hmLF, we wanted to further inspect the correlation between glycoform and ferroforms coexisting in human milk. Previous research has shown contradicting evidence on whether there is a relationship between glycosylation and iron-loading status of hmLF (1, 22, 35, 36). However, these studies relied only on biochemical assays that lack the systematic and native approach here utilized. To address this, we began by splitting the proteoform peaks based on their annotated iron-loading status (0, 1, or 2 Fe^3+^). To investigate whether any ferroform was preferentially enriched in hmLF, we first plotted the abundance of each peak measured at each specific iron-loading status (Figures 6a, 7a). This analysis confirmed the overall lower abundance of proteoforms with all 3 glycosites occupied (Figure 7a, *Σ* fractions) when compared to those proteoforms with just 2 sites occupied (Figure 6a, Σ fractions), but it also revealed no specific enrichment in particular ferroforms in terms of abundance in human milk. This was evidenced by the abundance of proteoforms with 0, 1, or 2 Fe^3+^ being similarly distributed (Figures 6a, 7a, Σ fractions).

Finally, to address the question of whether specific glycan features were associated with particular ferroforms, we calculated a combined glycan formula based on the individual glycans annotated for each peak. For example, for the base peak in F2 displayed in Figure 5d (labelled with an asterisk), the annotated glycans best explaining the measured mass were N4H5F2 and N4H5F2S1, resulting in a combined total glycan formula of N8H10F4S1. Using these combined glycan formulas, we examined whether specific glycosylation features, such as higher levels of branching (measured as the number of HexNAc residues), fucosylation, or sialylation were preferentially associated with the different ferroforms. This analysis did not reveal conclusive evidence for any glycosylation feature to be clearly correlated with the ferroforms (Figures 6b, 7b). However, we observed a trend suggesting that increased branching was associated with the *holo* (2-Fe^3+^) form of hmLF (Figures 6b, 7b, # of HexNAc). This trend was consistent both in proteoforms with 2 or 3 *N*-glycosylation sites occupied. These observations were consistent whether individual fractions were analyzed separately or collided into a “single fraction” (Figures 6b, 7b, Σ fractions). Furthermore, when analyzing the combined fractions, the trend of increased branching in the *holo* hmLF form was also supported by more of the glycans associated with *holo* ferroforms being mapped to triantennary glycans cores (Figures 6c, 7c).

## Discussion

The structural diversity of LF in human milk is central to its functional complexity and its crucial role in infant nutrition, immunity, and development. In this study, we characterized the glycosylation dynamics, proteoform heterogeneity, and iron-loading status of hmLF using hybrid MS approaches, consisting of bottom-up glycoproteomics, and IEX-native MS. These complementary approaches provide the first detailed and cohesive picture of hmLF’s temporal, site-specific glycosylation and ferroform heterogeneity. By analyzing both milk-derived phmLF and pbmLF, as well as by valuable donor-derived hmLF, our work provides a strong foundation supporting the diversity of LF, which is necessary for the development of LF-based commercial products for their use in infant and adult nutrition.

Our findings provide a detailed characterization of hmLF glycosylation over lactation, extending previous insights into its site-specific glycosylation dynamics up to 16 weeks postpartum. As previously shown, hmLF declined in concentration consistently in both donors, from ~1.5 mg/mL in week 1 to ~0.5 mg/mL in week 16 of lactation (see Lactotransferrin)^1^ (37), agreeing with the expected decline in concentration reported in literature (56, 57). The limited glycosylation observed at Asn642 (91 PSMs, ~1% of total evidence, Figure 1b) aligns with earlier reports of low site occupancy at this location (32, 58). Moreover, we consistently found the dominant proteoforms to be glycosylated at 2 out of 3 *N*-glycosylation sites (Figures 3, 5), probably at Asn156 and Asn497 as hinted by bottom-up glycoproteomics evidence (Figure 1). Notably, this observation strongly aligns with previous evidence, reporting that hmLF glycosylation occurs preferentially at two sites 85% (at Asn156 and Asn497), and less frequently at all 3 sites (10%), or at a single site (5%, at Asn497) (58). This resembles the observation in pbmLF by us (Figure 4) and others (55, 59, 60) of the co-existing of 2 main pbmLF forms differentiated by having either 4 or 5 out of 5 *N*-glycosites occupied. These differences in glycosite occupancy should be understood as resulting in proteoforms with different structural features, which may ultimately have a functional consequence, e.g., modulating iron binding affinity or iron release when this 3rd (or 5th for pbmLF) glycan present or absent. While this was outside the scope of this work, future research should be directed to understanding the functional implications of doubly or triply glycosylated hmLF proteoforms.

Notably, at Asn156 and Asn497, we identified a consistently small subset of dominant glycans shared between donors. These glycans exhibit striking stability over lactation, with a few dominant species increasing in abundance and reflecting a progressive curation of the initial glycan heterogeneity into a more stable, homogeneous profile over milk maturation (Figure 1c). This trend aligns with prior glycomic studies indicating a reduction in glycan complexity over time (33) and offers a more detailed, site-specific view of this process. Monitoring glycan patterns per site, we noted that branching remained consistent, while sialylation slightly decreased, and fucosylation slightly increased over lactation at Asn497 (Figure 1d). A similar trend was observed for Asn156 but without reproducible significance for both donors. Moreover, these findings are consistent with previous reports of increased fucosylation in mature milk (33) and highlight the dynamic nature of hmLF glycosylation as it adapts to the infant’s needs during lactation. By using a bottom-up glycoproteomics approach, this study complements existing glycomic evidence by revealing additional nuances of hmLF’s structural and functional diversity, such as glycan-to-site assignment and temporal site-specific information.

In this work, we also incorporated an IEX- native MS prefractionation strategy to obtain a complete, high-resolution picture of the proteoform landscape of hmLF. For hmLF, iron content drove the IEX elution behavior (Figure 3), Native MS done on 5 IEX obtained fractions, each with specific ferroforms preferentially enriched, revealed high heterogeneity in glycosylation and provided detailed characterization of glycoform modifications (Figure 5). In general, lactoferrin proteoforms glycosylated at all *N*-glycosylation sites were less abundant compared to those glycosylated at 2 of 3 sites for hmLF and 4 of 5 sites for bFL (Figures 3–5). However, for pbmLF the difference between the fully and partly glycosylated proteoforms was more muted (Figure 4b) than for hmLF. Compared to hmLF, pbmLF also displayed major differences in glycosylation. Within pbmLF’s 5 *N*-glycosylation sites, high-mannose glycans were predominantly found decorating the protein (Figure 4c). Similarly to hmLF, pbmLF also presented extensive glycan heterogeneity, as shown by the variety of glycoforms detected by native MS (Figure 4c). These observations strongly support the findings of a recent study which applied a similar approach to study bovine milk-derived LF glycan heterogeneity (34). It is important to note that human and bovine LF differ significantly in protein concentration (bovine milk-derived LF constitutes only 5% of the concentration found for LF in human milk) (61), in amino acid sequence identity (69%), and as noted by us and others, in glycosylation patterns (54). These structural, and consequently functional, differences observed between human and bovine milk-derived LF may have significant implications for their use in nutritional products.

In light of recent advancements aimed at producing LF with enhanced native *human-like* properties from bovine milk, alongside progress in the biotechnological production of recombinant bovine and human LF, it becomes evident that comprehensive MS-based analytics should be integrated into LF characterization. For instance, recombinant hmLF produced in yeast expression systems is becoming a preferred alternative to cow-milk-derived LF, yet various studies report notable differences compared to native human milk LF, particularly in terms of glycosylation profiles (32, 39, 54, 62, 63). Compared to native hmLF, recombinantly produced hmLF displays glycosylation that more closely resembles the high-mannose type observed here in bovine milk-derived LF (Figure 4), rather than the complex-type identified directly in human milk LF. Therefore, these advanced integrative MS-based approaches could serve as a steppingstone, not just for achieving thorough understanding of LF’s structural and functional diversity with regard to human nutrition, but also for supporting regulatory assessments that safeguard product quality in the industry.

Regarding the ferroforms, our findings did not provide conclusive evidence for any glycosylation trait to be clearly correlated (Figures 6b, 7b). However, we observed a trend suggesting that increased branching was associated with the *holo* (2-Fe^3+^) form of hmLF. This trend was consistent both in proteoforms with 2 or 3 glycosylation sites occupied. These observations were also consistent whether individual fractions were analyzed separately or collided into a “single fraction” (Figures 6b, 7b, Σfractions). Finally, when analyzing the combined fractions, the trend of increased branching in the *holo* hmLF form was supported by the increased annotation of triantennary glycans to peaks associated with 2 Fe^3+^ containing ferroforms (Figures 6c, 7c). This supported the observation that more extensively branched glycan structures may have a mild preference for association with *holo* hmLF, what calls for further investigation of specific functionalities of these lower abundant, more branched structures.

While this study deepens our understanding of the structural and functional complexity of human milk-derived LF, several limitations must be considered when interpreting the results obtained here. The donor cohort analyzed in this study was small, highlighting the need for validation in larger cohorts. Additionally, purified human milk and bovine milk LF samples may not fully represent native milk-derived LF, with potential biases introduced by the purification strategy itself. While our results suggested an association between specific glycosylation branching and *holo* hmLF, these observations require further investigation in larger datasets and call for functional studies to elucidate their biological significance. Finally, the absence of MS-based evidence for the presence of carbonate ions in Fe^3+^-bound hmLF must be addressed. Although carbonate is essential to stabilize Fe^3+^ (1, 25, 26), we found no evidence for its presence in gas-phase native LF spectra here recorded. We hypothesize that the lack of carbonate ions could be caused by ionization conditions, being the gas-phase stability of preloaded LF sufficient to hold Fe^3+^ in place.

To sum up, in this work we have applied hybrid MS approaches to characterize the intricate glycosylation dynamics and proteoform landscape of milk-derived LF. This protein displays overall a highly heterogeneous proteoform profile, aligning with its broad range of functions that are ultimately essential for infant nutrition and immune modulation. Analyzing donor-derived human milk, this work represents one of the first benchmarks of hmLF’s true structural diversity. This integrative analytical approach should prove useful for characterization of LF-derived nutritional and therapeutic products, as it could help to better mimic native hmLF glycoform and ferroform profiles. Future research with larger, more diverse cohorts and complemented with functional assays will help validate and contextualize these findings, further advancing the development of LF products for nutritional and therapeutic applications.

## Data Availability

The datasets presented in this study can be found in online repositories. The names of the repository/repositories and accession number(s) can be found below: http://www.proteomexchange.org/, PXD06503.

## Footnotes

1
https://milkprofiling.hecklab.com/
